# An unexpected evolution of symptomatic mild middle cerebral artery (MCA) stenosis: asymptomatic occlusion

**DOI:** 10.1186/1471-2377-11-154

**Published:** 2011-12-13

**Authors:** Giovanni Malferrari, Marialuisa Zedde, Gianni De Berti, Massimo Maggi, Norina Marcello

**Affiliations:** 1 Neurology Unit, Department of Neuromotor Physiology, Azienda Ospedaliera ASMN, Istituto di Ricovero e Cura a Carattere Scientifico, Viale Risorgimento 80, 42100 Reggio Emilia, Italy; 2 Department of Neuroradiology, Azienda Ospedaliera ASMN, Istituto di Ricovero e Cura a Carattere Scientifico, Viale Risorgimento 80, 42100 Reggio Emilia, Italy

## Abstract

**Background:**

The intracranial localization of large artery disease is recognized as the main cause of ischemic stroke in the world, considering all countries, although its global burden is widely underestimated. Indeed it has been reported more frequently in Asians and African-American people, but the finding of intracranial stenosis as a cause of ischemic stroke is relatively common also in Caucasians. The prognosis of patients with stroke due to intracranial steno-occlusion is strictly dependent on the time of recanalization. Moreover, the course of the vessel involvement is highly dynamic in both directions, improvement or worsening, although several data are derived from the atherosclerotic subtype, compared to other causes.

**Case description:**

We report the clinical, neurosonological and neuroradiological findings of a young woman, who came to our Stroke Unit because of the abrupt onset of aphasia during her work. An urgent neurosonological examination showed a left M1 MCA stenosis, congruent with the presenting symptoms; magnetic resonance imaging confirmed this finding and identified an acute ischemic lesion on the left MCA territory. The past history of the patient was significant only for a hyperinsulinemic condition, treated with metformine, and a mild overweight. At this time a selective cerebral angiography was not performed because of the patient refusal and she was discharged on antiplatelet and lipid-lowering therapy, having failed to identify autoimmune or inflammatory diseases. Within 1 month, she went back to our attention because of the recurrence of aphasia, lasting about ten minutes. Neuroimaging findings were unchanged, but the patient accepted to undergo a selective cerebral angiography, which showed a mild left distal M1 MCA stenosis.

During the follow-up the patient did not experienced any recurrence, but a routine neurosonological examination found an unexpected evolution of the known MCA stenosis, i.e. left M1 MCA occlusion. Neuroradiological imaging did not identify new lesions of the brain parenchyma and a repeated selective cerebral angiography confirmed the left M1 MCA occlusion.

**Conclusions:**

Regardless of the role of metabolic and/or inflammatory factors on the aetiology of the intracranial stenosis in this case, the course of the vessel disease was unexpected and previously unreported in the literature at our knowledge.

## Background

It has been known since several years that ischemic stroke related to large intracranial arteries steno-occlusion has a poor prognosis, if the vessel recanalization does not occur within 1 hour [1]. Indeed M1 MCA occlusion is recognized to be a very harmful condition with an unfavorable outcome. Several papers reported the rate and course of both symptomatic and asymptomatic intracranial stenosis and the dynamic progression of a symptomatic atherosclerotic MCA stenosis has been well described as a risk factor for stroke recurrence [2,3]. Conversely, only few data are available about asymptomatic intracranial occlusions and, at our knowledge, it is the first report of an asymptomatic main stem MCA occlusion, as evolution of a MCA stenosis.

## Case description

A 26 years-old woman came to our attention because of the abrupt onset of aphasia during her work (she was a lower school teacher). This symptom lasted several days with a progressive improvement during hospital staying and a final complete recovery. The patient arrived in the Emergency Department four hours after the onset of symptoms and an unenhanced brain CT did not show abnormalities. Urgent blood tests was also normal. The neurological evaluation reported a mild aphasia with prevalent anomic and paraphasic alterations, without motor deficits; the National Institute of Health Stroke Scale (NIHSS) score was 1.

**Figure 1:**
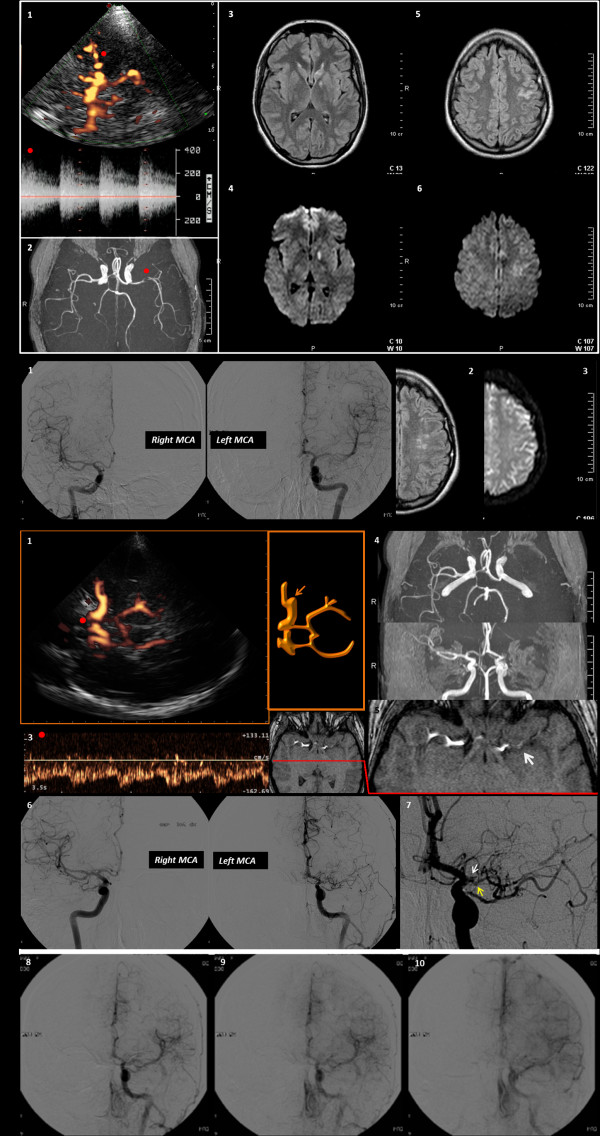
**Neuroimaging data of the patient**. The neurosonological and neuroradiological findings are shown. Row A refers to the first occurrence of stroke. 1. TCCS image with contrast agent from the left temporal bone window in the mesencephalic axial plane (power mode). The red dot points to the left M1 MCA, with the corresponding flow spectrum. 2. MRA with TOF reconstruction: the red dot points to the left MCA stenosis. 3. T2-weighted MRI with the caudate head lesion 4. Corresponding positive DWI-MRI 5. T2-weighted MRI with cortical sulcal damage 6. Corresponding positive DWI-MRI. Row B shows: 1. DSA images of right and left MCA, with the confirmed mild left M1-M2 stenosis. 2. T2-weighted MRI of the left hemisphere with the signs of the previous infarction. 3. corresponding negative DWI-MRI. Row C refers to the third neuroradiological and neurosonological control examinations. 1. TCCS image with contrast agent from the left temporal bone window in the mesencephalic plane (Power mode), showing the left main stem MCA stop, and the early MCA branch. 2. Corresponding schematic drawing. 3. The Doppler waveform of the left A1 ACA is showed (the red dot points on the corresponding vessel segment in the Power-mode TCCS image), and the increased flow velocity suggests a condition of flow diversion for contributing to the reperfusion of MCA territory. 4. TOF MRA reconstruction with absent signal from left M1 MCA. 5. MRA source image with the proximal left M1 MCA branch, nearest to the origin of the vessel, coursing along the silvian fissure (white arrow); see for comparison the normal right MCA in the same figure. 6. DSA image, showing a good correspondence with the neusonological findings, comparing the right and the left MCA. 7. Zoomed detail of the very early left MCA branching (yellow arrow) on DSA, just before the MCA occlusion, nearest to the carotid bifurcation (white arrow).8. 8, 9, 10 Temporal sequence of selective DSA with contrast injection in the left. 9. ICA, showing the slow reperfusion of the distal MCA territories by the early. 10. MCA branch and the contribution of the anastomosis with distal branches of the left ACA..

At this point an urgent neurosonological study was performed both in the extracranial and intracranial vessels, showing a normal appearance of the extracranial carotid and vertebral axis and a suspected left distal M1 MCA stenosis, by using unenhanced Transcranial Colour-Coded Sonography (TCCS). Therefore, because of the suboptimal temporal bone window, contrast-enhanced TCCS was performed by intravenous injection of sulphur hexafluoride (Sonovue^®^). It confirmed the presence of a left distal M1 MCA stenosis; moreover this finding was congruent with signs and symptoms of the patient. The ultrasound grading of the MCA stenosis was performed according to the Baumgartner's velocity criteria [4], and the stenosis was evaluated as ≥ 50% (Figure 1, row A). The left M2 MCA waveform did not show an abnormal pattern, also compared with the contralateral corresponding segment. The left A1 anterior cerebral artery (ACA) did not show signs of flow diversion and the posterior cerebral artery (PCA) waveform appeared normal.

Subsequent brain Magnetic Resonance Imaging (MRI) showed a left caudate head and temporal cortical ischemic damage and the Magnetic Resonance Angiography (MRA) confirmed the left MCA stenosis (Figure 1, row A).

The past medical history of the patient was significant only for a previous diagnosis of hyperinsulinemia, treated with metformine; overwheight is present (BMI 26). The diagnostic pathway included blood testing and instrumental evaluation to rule out a primary or secondary central nervous system vasculitis. An accurate anamnestic survey was performed about the previous occurrence of any event that could point to immunological and/or rheumatologic or infectious processes, with negative results. Blood samples were taken for screening of autoimmune diseases and common acquired or congenital thrombophylic factors or coagulation abnormalities, both with negative findings. The titration of autoantibodies did not show any abnormality, also including antiphospholipid and anti-beta2 glycoprotein 1 antibodies. Serological screening for borreliosis, herpesviruses (comprising Varicella-Zoster virus - VZV) and influenza viruses was not suggestive for a first or recurrent infection. Also the lymphocyte typing and the immunofixation electrophoresis of immunoglobulins were normal.

Antiplatelet and HMGCoA reductase inhibitor therapy was early started and a selective Digital Subtraction Angiography (DSA) was proposed, but the patient initially refused it.

After 20 days the patient was again evaluated because of the recurrence of a ten minutes-lasting episode of aphasia. Both neurosonological and neuroradiological examinations were repeated and their findings were unchanged; at this point the patient agreed to undergo selective DSA of the cerebral vessels. Therefore DSA was performed after 10 days. Meanwhile a dual antiplatelet therapy with acetyl salicylic acid and clopidogrel was suggested. The DSA findings (Figure 1, row B) was similar to TCCS and MRA results for the identification of the left distal M1 and M2 MCA branch stenosis, but the angiographic grading (50%) was lesser than the expected one.

In the following ten months no other event has been reported by the patient and she was phone called in order to undergo a contrast-enhanced TCCS as a follow-up test. TCCS was performed in second harmonic imaging with pulse inversion and sulphur hexafluoride (Sonovue^®^) 2.5 mL was injected by the right antecubital vein. A left M1-M2 MCA occlusion was diagnosed, with a large early branch, arising from the M1 MCA segment just before the stop (Figure 1, row C). This findings were really unexpected and therefore a brain MRI and MRA were performed, showing respectively the lack of new brain parenchymal lesions with a normal perfusional status, and an absent flow signal in the left M1-M2 MCA. Moreover this last finding was confirmed by a subsequently performed DSA, also viewing the early M1 MCA branch that vascularizes the distal MCA territories (row C of the Figure 1 lists the follow-up neurosonological and neuroradiological data).

In the following 16 months neurosonological control examinations overlapped the last findings and no neurological event was reported. Also neuropsychological testing was normal and the patient was treated with a Serotonine Selective Reuptake Inhibitor, because of an associated post-stroke depression.

## Discussion

This case, during its course, raises some relevant discussion points to be clarified during the clinical reasoning.

First, the infarction localization at the caudate nucleus is seldom documented in the literature in case of MCA stenosis [5,6]. This location of ischemic damage was found congruent with the MCA territory, because the lateral lenticulostriate arteries, arising from the M1 MCA, are known as main feeders of the caudate territory [7]. In this context the stroke can be classified, from the etiological point of view, as a parent artery disease (PAD) subtype of lacunar infarctions and the distal location of the MCA stenosis in the M1 segment agrees with the caudate site of the ischemic damage [8]. This subtype of lacunar stroke is caused by a large artery intracranial disease and it shares the prognosis with the large artery atherosclerosis subtype of stroke, i.e. a higher risk of recurrence than lacunar subtype caused by small vessel disease [9,10].

Second, the difference in the grading of the MCA stenosis between TCCS and DSA was somewhat surprising initially, and we have not a definite explanation about this single case from the literature data. It is possible that a high flow condition was present in order to maintain a sufficient perfusion downstream the stenosis and to sustain the collateral circulation. A similar situation has been seldom demonstrated in the post-acute phase of striato-capsular infarction, although it was considered a transient phenomenon [11]. The time interval between TCCS (Figure 1, row A) and DSA seems not to be responsible of this difference in grading, in our opinion, because a control TCCS was performed 24 hours after the DSA examination and the neurosonological grading was substantially unchanged.

The third but maybe more relevant point is the course of the vessel lesion, dramatically worsening, associated to a clinical silence. The asymptomatic course can help to explain the difference in grading, discussed above, because of the efficient recruitment of collateral channels, of which the high flow velocity of the left MCA at the site of the stenosis may be a marker. Several previous studies showed that intracranial atherosclerosis is an highly dynamic process, reporting both progression or regression by using neuroradiological [2] and neurosonological [3,12] techniques. The conclusion of all studies is that the progression of a symptomatic MCA stenosis is a strong predictor of a poor outcome and an independent predictor of stroke recurrence [13]. Therefore the evolution of the present case is really unexpected, both from the vascular perspective and from the clinical one.

In the case series of Kern and colleagues [14] only two patients were reported to have an asymptomatic MCA occlusion, but without information about the previous condition of the vessel. The evolution in the present case was quite different, and the same study [14] concluded that "progression from stenosis to occlusion was not observed" in 102 consecutive patients with atherosclerotic MCA disease.

The evolution of the MCA stenosis in our case was not previously described in the literature, at our knowledge, not only for intracranial atherosclerotic stenosis but also for other causes of intracranial vessel disease. Diagnostic investigations about the causes of the MCA stenosis in our patient were substantially negative and the neuroradiological findings were not pathognomonic for vasculitis, dissection or a moiamoia disease, although this last condition can be very challenging in the initial phase and a longer follow-up could allow to identify the involvement of other intracranial vessels. At the moment, however, a clear definition about the causes of the MCA stenosis has not been reached in our case.

## Conclusions

It is possible that inflammatory and metabolic factors, potentially related to each other, have played a role in the evolution of the vessel history, but both vascular and clinical course of this case are previously unreported in the neurological literature at our knowledge.

## Consent

Written informed consent was obtained from the patient for publication of this case report and any accompanying images.

## Competing interests

The authors declare that they have no competing interests.

## Authors' contributions

GM revised the draft and performed the first ultrasound evaluation; MZ wrote the draft and performed the follow-up of the patient; GDB revised the neuroradiological examinations and co-revised the draft; MM performed the neuroradiological follow-up and co-revised the draft; NM revised the draft.

All authors read and approved the final manuscript.

## Pre-publication history

The pre-publication history for this paper can be accessed here:

http://www.biomedcentral.com/1471-2377/11/154/prepub
